# Supplementary material for: Type I Cystatin Derived from Cysticercus pisiformis—Stefins, Suppresses LPS-Mediated Inflammatory Response in RAW264.7 Cells
Source: Microorganisms. 2024 Apr 24;12(5):850. doi: 10.3390/microorganisms12050850 (PMC11123757; doi:10.3390/microorganisms12050850)
Supplement: Supplementary file 1 [file microorganisms-12-00850-s001.zip › microorganisms-2918007-supplementary.pdf]

microorganisms

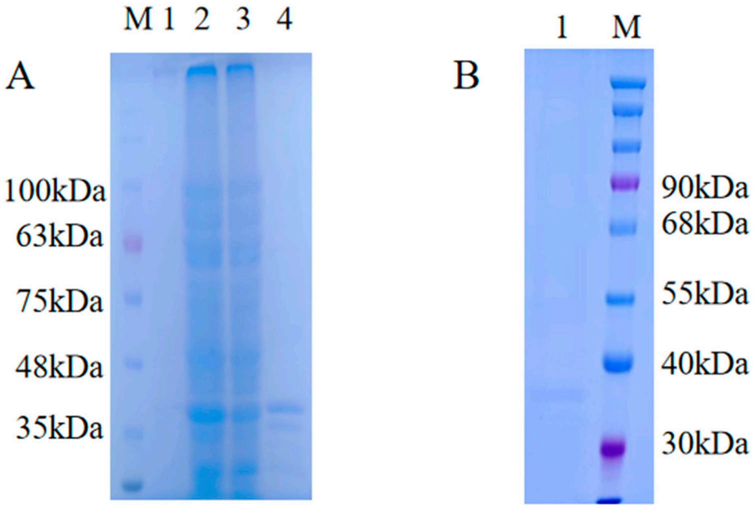

Figure S1. Expression and purification of recombinant CpStefin protein. **A**. The expression of recombinant CpStefin protein. M: protein Marker; 1: uninduced control; 2: total supernatant protein after IPTG induction; 3: total protein of whole liquid after IPTG induction; 4: total protein of precipitation after IPTG induction. **B**. The purification of recombinant CpStefin protein. M: protein Marker; 1: purified CpStefin recombinant protein.
